# Blue-light reception through quaternary transitions

**DOI:** 10.1038/s41598-017-01497-7

**Published:** 2017-05-03

**Authors:** Christopher Engelhard, Ralph P. Diensthuber, Andreas Möglich, Robert Bittl

**Affiliations:** 1 Fachbereich Physik, Institut für Experimentalphysik, Freie Universität Berlin, 14195 Berlin, Germany; 2 Biophysikalische Chemie, Institut für Biologie, Humboldt-Universität zu Berlin, 10115 Berlin, Germany; 3 Lehrstuhl für Biochemie and Research Center for Bio-Macromolecules, Universität Bayreuth, 95440 Bayreuth, Germany

## Abstract

Sensory photoreceptors absorb light via their photosensor modules and trigger downstream physiological adaptations via their effector modules. Light reception accordingly depends on precisely orchestrated interactions between these modules, the molecular details of which often remain elusive. Using electron-electron double resonance (ELDOR) spectroscopy and site-directed spin labelling, we chart the structural transitions facilitating blue-light reception in the engineered light-oxygen-voltage (LOV) histidine kinase YF1 which represents a paradigm for numerous natural signal receptors. Structural modelling based on pair-wise distance constraints derived from ELDOR pinpoint light-induced rotation and splaying apart of the two LOV photosensors in the dimeric photoreceptor. Resultant molecular strain likely relaxes as left-handed supercoiling of the coiled-coil linker connecting sensor and effector units. ELDOR data on a photoreceptor variant with an inverted signal response indicate a drastically altered dimer interface but light-induced structural transitions in the linker that are similar to those in YF1. Taken together, we provide mechanistic insight into the signal trajectories of LOV photoreceptors and histidine kinases that inform molecular simulations and the engineering of novel receptors.

## Introduction

Throughout nature, organismal behaviour and physiology are widely adapted in response to light. To this end, spatial and temporal information contained in incident light is captured by sensory photoreceptors that comprise photosensor and effector modules^1^. Among the different photoreceptor classes, the blue-light-sensing light-oxygen-voltage (LOV) proteins^2, 3^ are remarkable for the wide array of associated effectors^4–6^ and biological processes they regulate, e.g., phototropism, chloroplast movement, stress response, and circadian rhythm^4, 7–9^. This diversity notwithstanding, all known LOV photosensors share the highly conserved Per-ARNT-Sim (PAS) fold^6^ and a flavin chromophore, either flavin mononucleotide (FMN) or flavin adenine dinucleotide. In the canonical, well-characterised LOV photocycle, absorption of blue light by the chromophore promotes formation of a thioether bond^10^ between atom C4a of the flavin isoalloxazine and atom Sγ of an adjacent, strictly conserved cysteine residue. Thioadduct formation likely involves a radical-pair intermediate^11, 12^ and is accompanied by protonation of the flavin N5 atom. Adduct formation is fully reversible, and scission of the thioether bond occurs thermally in a base-catalyzed process^13^. Although the generation of the thioadduct was originally considered essential for light-dependent signalling, we recently demonstrated that N5 protonation alone suffices^12^. Changes in N5 protonation trigger subsequent rearrangements of hydrogen bonds throughout the LOV photosensor, with a conserved glutamine residue in particular engaged in coupling photochemical events within the flavin chromophore to the LOV protein scaffold. Light-induced structural and dynamic perturbations propagate through the LOV domain^8^ and ultimately to the effector, thereby modulating its biological activity^4, 7, 8^. Within the paradigmatic LOV2 photosensor from *Avena sativa* phototropin 1, light-induced transitions culminate in reversible unfolding of an ancillary α helix, denoted Jα, immediately C-terminal to the LOV core^14^. By contrast, the molecular details of signal propagation in most other LOV photosensors and of transmission to variable effectors underlying the extraordinarily broad range of biological responses remain largely elusive.

**Figure 1:**
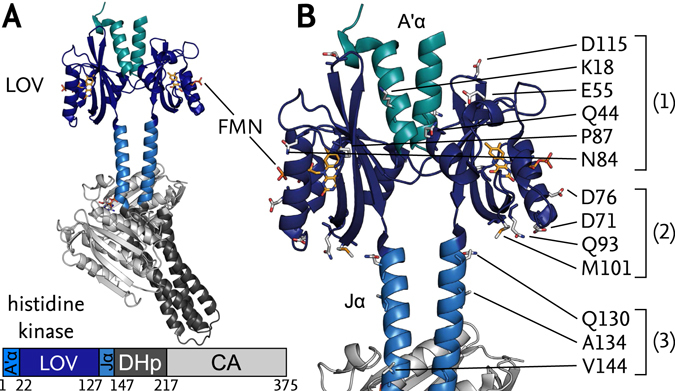
(**A**) Structure of YF1 with a LOV photosensor dimer, consisting of A’α helices (cyan) and LOV core domains (dark blue, FMN chromophores in yellow), linked to the histidine kinase effector, consisting of DHp (dark grey) and CA subdomains (light grey), by the coiled-coil Jα linker (light blue). (**B**) Close-up of LOV photosensor and linker region with the residues targeted for spin labelling marked as sticks. Positions were chosen within: (1) the upper halves of the LOV domains; (2) the lower halves; and (3) the Jα linker.

The modular architecture of sensory photoreceptors enables the engineering of novel, light-responsive receptors via recombination of photosensor and effector units^15^. As a case in point, we generated the chimeric photoreceptor YF1^16^ by linking the *Bacillus subtilis* YtvA (*Bs*YtvA) LOV photosensor domain to the histidine kinase effector of *Bradyrhizobium japonicum* FixL (*Bj*FixL), consisting of DHp (dimerisation and phospho-acceptor histidine) and CA (catalytic) subdomains. In darkness, YF1 phosphorylates the cognate response regulator *Bj*FixJ but following blue-light absorption net histidine kinase activity is strongly repressed^17^. When phosphorylated, *Bj*FixJ drives expression from the specific *Bj*FixK2 promotor thus allowing facile readout of light-dependent YF1 activity^18^. The high-resolution structure of homodimeric YF1 in its dark-adapted state (PDB entry 4GCZ^19^) (Fig. 1A) revealed its two LOV photosensor domains to be connected to the dimeric DHp domain through a continuous α-helical coiled coil, denoted Jα, as earlier stipulated based on biochemical and sequence evidence^16^. A second, coaxial coiled coil, denoted A’α, is wedged in between the two LOV photosensors. Catalytic activity and regulation by light strongly depended on the length of the Jα linker with a pronounced seven-residue (heptad) periodicity. Pairs of YF1 variants that differed in linker length by multiples of seven residues usually showed similar light response; by contrast, insertion of single residues into the coiled coil could invert the signal polarity, i.e. reprogram YF1 to become light-activated instead of light-repressed^16, 18^. Based on these observations, we posited that signal transduction in YF1 depends on rotary or torque motions in the Jα coiled-coil linker. Inversion of signal polarity could not only be achieved by linker-length modifications but also by exchanges of single residues within the A’α helices at the interface between the two LOV domains of YF1, e.g., in the variants D21V and H22P^20^.

To assess signal transduction in the parental *Bs*YtvA photoreceptor, we had previously crystallized its isolated LOV photosensor dimer in its dark-adapted state and solved its structure (2PR5^21^). Diffraction data on blue-light-exposed and rapidly cryo-cooled crystals (2PR6) implied that in the light-adapted state the two LOV domains slightly rotate against another compared to the dark-adapted state. However, the relevance of these motions for signal transduction in full-length *Bs*YtvA and YF1 was unclear since experiments were subject to packing effects within the crystal lattice and since the *Bs*YtvA construct was N-terminally truncated lacking the A’α helices, thus resulting in a compromised dimer interface.

Despite the wealth of information on the dark-adapted states, the molecular aspects underlying light-dependent signal transduction in YF1 and *Bs*YtvA remain to be elucidated, largely due to a lack of detailed structural information on the light-adapted states which are only transiently populated. We hence resorted to electron paramagnetic resonance (EPR) spectroscopy as a technique that can resolve these states in solution. To this end, both the dark-adapted and light-adapted states of YF1 were probed by pulsed electron-electron double resonance (ELDOR)^22, 23^ distance measurements in conjunction with site-directed spin labelling (SDSL)^24^. Resultant pair-wise distance constraints allowed triangulation of the quaternary structure of YF1 and revealed that in the light-adapted state the two LOV photosensors are slightly splayed apart and rotated against each other relative to the dark-adapted state. Signal propagation to the histidine kinase effector is likely achieved via left-handed supercoiling of the Jα coiled coil. In the signal-inverted H22P variant, the LOV dimer interface was drastically altered yet qualitatively similar quaternary rearrangements were induced by light. Not only do our data bear on signal transduction in the widespread family of sensor histidine kinases, but also they inform the engineering of optogenetic actuators.

## Results

### Site-directed spin labelling of YF1

**Figure 2:**
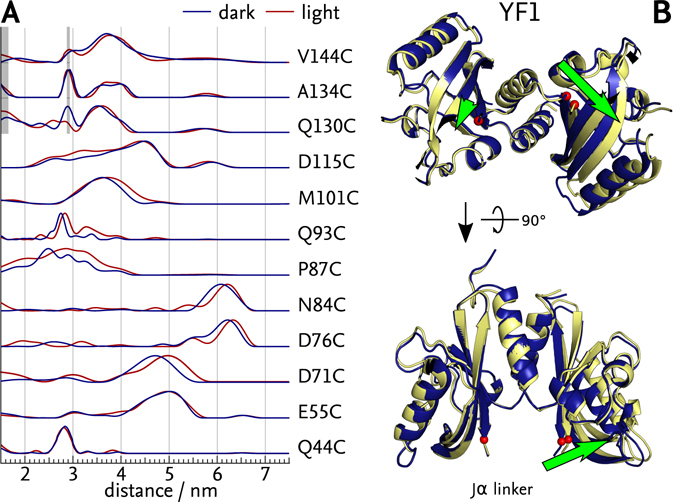
ELDOR-based structural model of light-induced transitions in YF1. (**A**) Distance distributions of dark-adapted (blue) and light-adapted (red) states derived from ELDOR experiments (Suppl. Figs S2 and S3). Areas shaded gray indicate artefacts arising from proton and deuteron modulations. Labels positioned near the A’α helices (Q44C, E55C) and in the linker (Q130C, A134C and V144C) showed no change upon illumination, others showed shifts to larger distances. (**B**) Transition from dark-adapted (blue) to light-adapted (yellow) state as modelled by ENM including the P87C constraint. Predominant structural changes are marked by green arrows, and the attachment sites for the Jα linker are indicated by red spheres.

To pinpoint structural rearrangements upon light absorption in YF1, we selected thirteen residue positions throughout the LOV domain and the Jα coiled-coil linker for SDSL based on solvent accessibility, suitable distances for ELDOR and proximity to regions of interest. The positions can be assigned to three groups (Fig. 1B): (1) residues K18, Q44, E55, N84, P87 and D115 in the upper half of the LOV photosensor (in the YF1 orientation in Fig. 1); (2) D71, D76, Q93 and M101 in the lower half; and (3) Q130, A134 and V144 within said Jα linker. YF1 variants with one of the indicated residues altered to cysteine were produced and labelled with (1-Oxyl-2,2,5,5-tetramethylpyrroline-3-methyl) methanethiosulfonate (MTS). To avoid the additional complexity arising from multi-spin samples, YF1 variants were limited to single cysteine replacements (see Supporting Information (SI) for details).

The structural and functional integrity of these variants was assessed in a three-stage process: First, the impact of the cysteine replacements on light-dependent signal transduction was probed in *E. coli* (see SI)^18^. With the exception of variant K18C, in none of the other variants introduction of a cysteine significantly impaired histidine kinase activity or light responsiveness (Suppl. Fig. S1A). Second, the function of the purified and MTS-labelled proteins was tested (Suppl. Fig. S1B)^12^. Most variants retained YF1-like activity and light regulation even after attachment of the spin label, except for Q93C and Q130C which lost any light responsiveness and were constitutively locked in the kinase-active and kinase-inactive states, respectively, regardless of illumination. Variant V144C displayed impaired light responsiveness compared to YF1. Third, to test that mutant variants still structurally corresponded to the original YF1 protein, the measured ELDOR data in the dark-adapted state were validated against the dark-adapted crystal structure 4GCZ as discussed below. Only variants that passed all three validation steps were used in further analysis.

### ELDOR distance measurements in dark- and light-adapted YF1

To determine interatomic distances between the labelled positions in YF1, we first recorded ELDOR traces on dark-adapted samples. The time evolution signal of all variants showed clear modulations indicative of two spatially close, interacting spin species (Suppl. Fig. S2). Following background correction (Suppl. Fig. S3), distance probability distributions *p*(*r*) were determined by Tikhonov regularisation (Fig. 2A). For positions Q44C, E55C and N84C situated in the upper half of the LOV photosensor dimer (cf. Fig. 1), the *p*(*r*) distributions showed single dominant, fairly narrow distance peaks centred at 2.8 nm (Q44C), 5.0 nm (E55C) and 6.1 nm (N84C), respectively (Fig. 2A, blue lines). The two remaining positions within the upper half of the LOV photosensor dimer featured broader distance distributions, centred at around 2.7 nm for P87C and with several distance contributions between 2.0 nm and 5.0 nm for D115C. Positions within the lower half of the LOV photosensor dimer also showed narrow distance distributions centred at 4.7 nm for D71C, 6.2 nm for D76C, 2.7 nm for Q93C and 3.6 nm for M101C. Of the positions within the coiled-coil linker, Q130C alone showed good signal-to-noise ratio (SNR) but a rather broad probability distribution of distances between 1.5 nm and 4.2 nm. By contrast, both A134C and V144C had very low SNR and were heavily affected by spurious distance contributions arising from proton and deuteron artefacts (Fig. 2A, shaded grey). Nonetheless, dominant distances of 3.7 nm (Q130C), 3.8 nm (A134C) and 3.7 nm (V144C) could be identified.

To assess whether introduction of the spin labels into YF1 caused detrimental structural perturbations (cf. above), we validated the *p*(*r*) distributions obtained by ELDOR against the YF1 crystal structure 4GCZ^19^ (see SI and Suppl. Fig. S4). Overall, for positions within the LOV photosensor dimer the distance distributions calculated from the crystal structure well fit the experimentally determined ones. Merely in variants P87C and Q93C, the calculated distances substantially overestimated the experimental ones. However, in the case of P87C, a subpopulation of the calculated MTS rotamer conformations can well describe the observed distribution. All three positions within the coiled-coil linker yielded distributions incompatible with the crystal structure and indeed, with a coiled-coil conformation of the linker. These findings largely coincide with the above functional assays that had shown these variants to be dysfunctional. Consequently, ELDOR data on the spin-labelled Q93C, Q130C, A134C and V144C variants were excluded from further analysis. On the whole, the introduction of site-specific paramagnetic spin labels at several positions throughout the core LOV photosensor mostly preserved structure and light-regulated function but the introduction of labels into the Jα coiled coil invariably disrupted them.

We also recorded ELDOR traces on all variants in their light-adapted state following saturating irradiation with blue light (Suppl. Figs S2 and S3). The derived distance probability distributions *p*(*r*) were largely unaffected by illumination for positions Q44C, E55C and D115C within the upper half of the LOV photosensors and for Q130C, A134C and V144C within the Jα coiled coil (Fig. 2A, red lines). By contrast, all positions within the lower half of the LOV photosensor, D71C, D76C, N84C, P87C, Q93C and M101C, exhibited a collective shift by between 0.1 and 0.3 nm towards larger distances relative to the dark-adapted state. Even prior to any detailed analysis, the location of the spin labels for which distance increases were detected, suggested light-induced tilting apart of the lower halves of the two LOV photosensor units.

### Modelling light-induced structural transitions in YF1

To gain a molecular understanding of the conformational transitions YF1 undergoes during light reception, we modelled the structure of its light-adapted state on the basis of the dark-adapted crystal structure 4GCZ and the ELDOR data. The centre distances of the dominant features in the ELDOR-derived *p*(*r*) distributions were weighted by the width of these distributions and were used as experimental constraints (see SI). As these constraints are few in number and the differences in *p*(*r*) between dark-adapted and light-adapted state are small in amplitude, care must be exerted to ensure that the modelling algorithm indeed captures transitions genuinely relevant to signal transduction. To this end, we employed two separate modelling algorithms, i) an elastic network model (ENM) implemented in MMM^25^; and ii) a constrained rigid-body docking algorithm (RBD) implemented in mtsslDock^26^, to extract conformational transitions that remained consistent and were hence algorithm-independent.

To account for and filter out potential systematic deviations between experimental data and modelled structures, we conducted separate simulations not only for the light-adapted state but also for the dark-adapted state. In this way, the overall quality of the modelling process can be assessed by comparing the dark-state model to the actual dark-state crystal structure 4GCZ. Regardless of whether the constraint for P87C was included or not, both modelling algorithms produced reasonable approximations for the dark-adapted state without large deviations from the crystal structure. Furthermore, all modelling runs yielded mutually consistent results that represented the crystal structure about equally well, with RMSD values between of 0.2 nm–0.4 nm.

When simulating the light-adapted state, structures derived from ENM produced a better representation of the observed distance changes than those generated by RBD. This indicates that the structural changes cannot be explained exclusively by a movement of the LOV domains relative to each other, but include small conformational changes within the LOV monomers as well. All simulations for the light-adapted state produced mutually consistent structural models that subtly differed from those of the dark-adapted state. Irrespectively of which algorithm was used and whether the P87C constraint was considered, qualitatively closely similar results were obtained (Suppl. Fig. S5). Compared to the respective dark-state models (Fig. 2B, blue), the models for the light-adapted state (Fig. 2B, yellow) revealed a rotation of the two LOV photosensor units against each other by about 7° and a concomitant tilting apart of their lower halves. Accompanying these rearrangements, the attachment sites for the C-terminal Jα helices moved apart by around 0.25 nm and changed their relative angular orientation (Fig. 2B, green arrows and Suppl. Fig. S5). While the amplitude of these shifts varied between models, the general motif of outward movement was fully consistent.

### ELDOR distance measurements in YF1 H22P with inverted signal response

We extended the ELDOR-based interrogation of functionally relevant structural transitions governing light reception to the H22P variant of YF1 which possesses inverted signal response despite only differing in the identity of one residue^19^. As a conceivable hypothesis, one may hence assume that the light-adapted state of H22P structurally corresponds to the dark-adapted state of YF1, and *vice versa*. To verify or falsify this hypothesis, we selected for spin attachment the same positions within the LOV photosensor as above for YF1. Merely positions K18, A134 and V144 which failed to yield interpretable distance constraints for YF1 were excluded right away; moreover, upon spin labelling, the H22P:Q44C variant could not be produced with sufficient incorporation of the FMN chromophore. Functional assays, performed as before, indicated that introduction of a cysteine residue in the H22P variant was well tolerated at all desired positions, and light-regulated function was largely retained (Suppl. Fig. S1C). Subsequent spin labelling completely abolished catalytic activity for the H22P:Q130C variant and impaired it to varying extent for H22P:D76C and H22P:M101C (Suppl. Fig. S1D). Unexpectedly, introduction of the spin label at position Q93C preserved light-regulated function in the H22P background where it had failed to do so in YF1.

**Figure 3:**
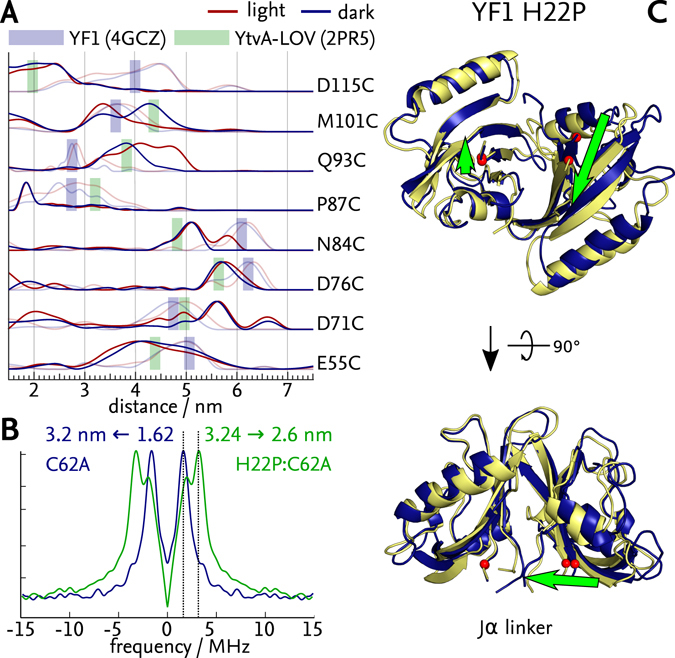
ELDOR-based structural model of light-induced transitions in YF1 H22P. (**A**) Distance distributions of dark-adapted (blue) and light-adapted (red) states derived from ELDOR data (Suppl. Figs S6 and S7). For reference, the distance distributions for YF1 from Fig. 2A are shown in half tones. Green rectangles indicate the mean distance of labels calculated for the *Bs*YtvA LOV structure 2PR5, blue ones those for YF1 4GCZ. Overall, the distances for the dark-adapted and light-adapted states of H22P do not match those of YF1 but those calculated for *Bs*YtvA LOV. (**B**) Dipolar spectrum resulting from ELDOR traces of light-exposed YF1 C62 A (blue) and YF1 C62A:H22P (green), showing a clear shift towards higher frequencies in YF1 C62A:H22P. The inter-flavin distance calculated for YF1 C62A:H22P is smaller by about 0.5 nm than that of YF1 C62A. (**C**) Transition from dark-adapted (blue) to light-adapted (yellow) state as modelled by ENM including the P87C constraint. Predominant structural changes are marked by green arrows, and the attachment sites for the Jα linker are indicated by red spheres.

Analogously to before, ELDOR experiments were conducted for the spin-labelled H22P variants in their dark-adapted states (Suppl. Figs S6 and S7). Strikingly, the *p*(*r*) distributions corresponded to neither those of dark- nor light-adapted YF1 (Fig. 3A). Rather, variants H22P:E55C, H22P:D76C, H22P:N84C, H22P:P87C and H22P:D115C all showed distributions with shorter distances than in YF1, with the difference being smallest in H22P:D76C (approx. 0.4 nm shorter than the dark-adapted state of YF1) and largest in H22P:D115C (1.5 nm shorter). By contrast, variants H22P:D71C, H22P:Q93C and H22P:M101C instead showed *p*(*r*) distributions centred at distances larger by about 0.8–1.0 nm than observed in dark-adapted YF1. ELDOR measurements on the light-adapted H22P variants showed that changes in the distance distributions induced by blue light were more complex than in YF1 (Fig. 3A, red). Some variants, e.g., H22P:E55C and H22P:M101C, exhibited shifts towards shorter distances, but others, e.g., H22P:Q93C and H22P:D76C, showed shifts towards longer distances. Overall, the absolute differences in *p*(*r*) between dark-adapted and light-adapted H22P were larger than those above observed for the YF1 variants.

### Modelling light-induced structural transitions in YF1 H22P

The analysis of light-induced structural transitions in YF1 H22P was complicated by the lack of a high-resolution structure of this variant. ELDOR data for several spin attachment sites, e.g., D115C, N84C and D76C, yielded interatomic distances too short to be compatible with the dimer interface observed in the YF1 crystal structure 4GCZ (cf. Fig. 1). In particular, the presence of the two N-terminal A’α helices evidently imposes a minimum distance of separation between the two LOV photosensors and thus most spin label pairs. We hence wondered whether the non-conservative exchange of histidine 22 to proline disrupted said A’α helices and allowed for closer approach of the LOV photosensors than seen in the 4GCZ structure. To test this hypothesis, we modelled the interatomic distances on the basis of the dark-adapted structure of a truncated *Bs*YtvA LOV construct (PDB: 2PR5, residues 20–147)^21^. Notably, in this structure the N-terminal A’α helices were cut off, thus yielding a dimer interface in which the two LOV photosensors directly associate via their β-sheets. Revealingly, the experimentally determined distances in H22P (Fig. 3A, blue) were in much better agreement with those expected on the basis of the *Bs*YtvA LOV structure 2PR5 (Fig. 3A, green shaded bars) than with those calculated for the YF1 structure 4GCZ (Fig. 3A, blue shaded bars), an exception again being H22P:P87C, which showed a significantly shorter distance than expected. We thus concluded that in YF1 H22P the N-terminal A’α helices are not tucked in between the LOV photosensors but rather project outward and might be unfolded.

We sought additional evidence for the altered dimerization interface in H22P and thus investigated the two YF1 variants C62A and H22P:C62A. Replacement of the conserved cysteine 62 by alanine prevents the formation of the cysteinyl-C4a-adduct, but allows photoreduction of flavin to the neutral semiquinone (NSQ) radical upon illumination^27^. The NSQ radical can then be used as an intrinsic spin probe for distance measurements^12, 28, 29^. As the spin density is mainly centred on the N5 and C4a atoms of flavin^30, 31^, corresponding ELDOR experiments generally yield narrow distance distributions not prone to the positional uncertainty introduced by the flexibility of the extrinsic MTS spin label. With the FMN chromophores situated on either side of the LOV dimer interface, their distance of separation thus unambiguously demonstrates the presence or absence of the N-terminal A’α helices. Both for C62A and H22P:C62A, the ELDOR measurements showed strong modulations, indicative of narrow distance distributions (Fig. 3B). Crucially, the mean distance of (2.6 ± 0.1) nm determined for H22P:C62A (Fig. 3B, green) was in quantitative agreement with the (2.5 ± 0.1) nm separation of the flavin chromophores in the *Bs*YtvA LOV structure 2PR5 that lacks the A’α helices. By contrast, the inter-flavin distance in the YF1 structure 4GCZ with intact A’α helices amounts to (3.0 ± 0.1) nm which matches the experimentally determined inter-flavin distance of (3.2 ± 0.1) nm for the C62A variant (Fig. 3B, blue). It should be noted that the photo-reduced NSQ radical states of the C62A and H22P:C62A variants functionally correspond to the light-adapted, thioadduct states of the respective cysteine-containing variants as recently demonstrated^12^.

To arrive at a molecular view of light-induced transitions in the signal-inverted H22P variant, we modelled structural changes based on the *Bs*YtvA LOV structure 2PR5 which is broadly consistent with all EPR-derived distance constraints for the dark-adapted state of H22P (cf. Fig. 3A and B). Nonetheless, we caution that this represents but an approximation and that results should hence be interpreted in qualitative rather than quantitative terms. Modelling was again performed with and without the H22P:P87C constraint (see SI and Suppl. Fig. S8). Both models for the dark-adapted state of H22P agreed well with the *Bs*YtvA LOV structure 2PR5, especially in the β-sheet region, with an overall RMSD of 0.26 nm, thereby lending strength to our using the ENM model based on 2PR5 (see Suppl. Fig. S9) as a template for the dark-adapted state of H22P (Fig. 3C, blue). In comparison, the model of the light-adapted state of H22P displayed a rotation of the two LOV photosensors against each other by about 6°, reminiscent of the light-induced structural transition observed for YF1, although the rotation axis is different. Again, this movement translated into changes of relative position and orientation of the Jα attachment sites (Fig. 3C, green and Suppl. Fig. S8). Intriguingly, for H22P the Jα attachment sites were closer to another in the light-adapted state in comparison to the dark-adapted state, which is opposite to the above findings for YF1.

## Discussion

### Light-induced pivoting of LOV photosensors underpins signal transduction in YF1

**Figure 4:**
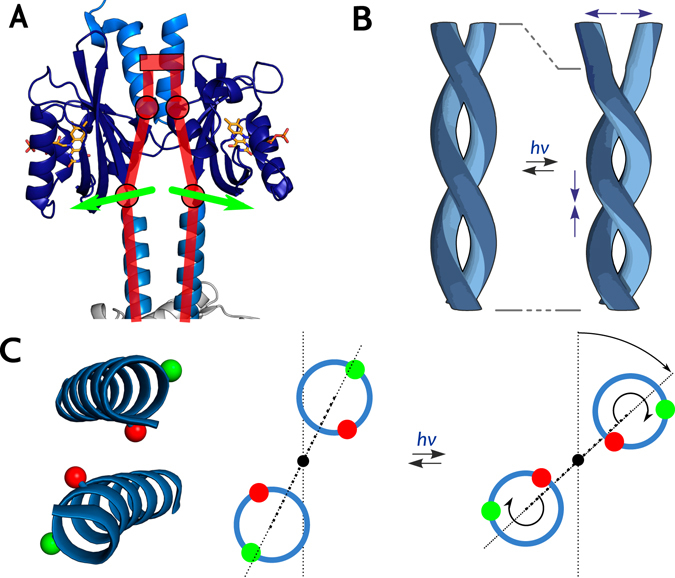
Schematic of the transition from dark-adapted to light-adapted state of YF1 and proposed signal transduction mechanism. (**A**) The outward tilt of the LOV domains in a hinge-like motion (red) causes separation of the N-termini of the coiled-coil linker (green arrows). (**B**) Separation of the N-termini would induce torque and left-handed supercoiling of the coiled-coil linker. (**C**) As a result, the C-termini of the linker helices (inset) would be rotated relative to the C2 axis of the coiled coil, causing both a rotation of the LOV dimer relative to the histidine kinase effector (dotted line) and an angular movement of the helices themselves. To illustrate the resultant angular displacement, within each helix two positions separated by one residue are highlighted in green and red, respectively.

Using SDSL and ELDOR spectroscopy, we triangulated the structures of the dark-adapted and light-adapted states of YF1. Pair-wise distances collectively increased upon illumination for spin labels situated in the lower half of the LOV photosensor dimer (in the orientation shown in Fig. 1), but they stayed largely constant in the upper half. Even in the absence of more elaborate evaluation, these observations directly implicate tilting apart of the two LOV monomers as a dominant feature of the light-induced structural transition (Fig. 4A). Structural modelling constrained by the ELDOR-derived experimental distance data consistently demonstrated that the tilting is accompanied by a slight rotation of the two LOV photosensors relative to each other. In combination, these structural rearrangements result in an increased distance and change in relative orientation of the attachment sites for the Jα helices at the LOV C-termini. Thus, the LOV domains apparently act as a lever on the linker region, with their N-termini as the hinge (Fig. 4A). As attachment of MTS spin labels within Jα invariably led to disruption of structure and light-regulated function, we could not directly monitor events and associated distance changes in this region, let alone within the DHp/CA effector. However, as experiments were performed in the context of the intact full-length protein, the ELDOR data report on functionally relevant, light-induced structural transitions that facilitate signal transduction in YF1. Intriguingly, a parallel investigation by X-ray solution scattering demonstrates essentially the same light-induced transitions within the isolated LOV photosensor dimer^32^. Moreover, transient grating studies on the isolated *Bs*YtvA LOV photosensor recently revealed an increase in the hydrodynamic radius upon blue-light absorption^33^ which is fully consistent with the light-induced pivot transition observed presently by EPR spectroscopy. We thus deem it likely that this structural mode also underpins photoreception in full-length *Bs*YtvA.

The present data also provide insight into downstream signal propagation through the Jα coiled-coil linker to the histidine kinase effector module. Light-driven splaying apart of the lower halves of the LOV photosensor units causes separation of the N-termini of the Jα helices (Fig. 4A). Resultant tilting apart of the Jα helices induces molecular strain and affects the stability of the coiled-coil linker. Relaxation of strain could induce torque movements and left-handed supercoiling (i.e. overwinding) of the coiled-coil linker (Fig. 4B) which we previously invoked as the molecular mechanism of signal propagation^16^. Such alterations in helical orientation and supercoiling can readily be relayed to the histidine kinase DHp domain as it forms a continuous helical bundle with the Jα linker^19^. In particular, the eponymous active-site histidine is embedded in this bundle, and even subtle changes in its accessibility may greatly affect catalytic activity. Although speculative, the torque model is further supported by three completely independent lines of evidence. First, recent work on the DesK histidine kinase from *Bacillus subtilis* reveals that in its phosphatase state (corresponding to light-adapted YF1) the DHp helices are rotated such that the active-site histidines are sequestered in the interior of the antiparallel four-helix bundle of the DHp domain^34^. Negative supercoiling of the Jα linker helices as we propose would prompt precisely such angular reorientation of the DHp helices and the catalytically important histidine residues. Second, an implicit prediction of the model is that changes in supercoiling of the coiled-coil linker induce global rotation of the LOV photosensor dimer relative to the DHp/CA effector (Fig. 4C) which is indeed observed by X-ray solution scattering data on full-length YF1 (Berntsson, Diensthuber, Möglich, Westenhoff, under revision). Third, recent data on scores of YF1 variants differing in linker composition revealed that light-regulated function strictly depends on discrete linker lengths^35^. Light-repressed histidine kinase activity as in the original YF1 predominantly required linkers of 7*n* residues length but signal-inverted, light-enhanced activity was found for linkers of 7*n* + 1 residues. Introduction of an additional residue in the 7*n* + 1 variants evidently induces a register shift in the coiled coil, strongly arguing that catalytic activity and regulation by light are primarily governed by angular orientation (Fig. 4C). Notably, in the dark-adapted state the 7*n* and 7*n* + 1 registers are associated with high and low kinases activities, respectively. By contrast, in the light-adapted state, it is the 7*n* + 1 register that is associated with high kinase activity, arguably because angular orientation changed such that it resembles that of the 7*n* register in the dark-adapted state. Left-handed supercoiling as proposed here and supported by solution scattering (Berntsson, Diensthuber, Möglich, Westenhoff, under revision) would lead to exactly that type of structural rearrangement.

### Similar signalling mechanism despite inverted signal response and altered dimer interface in the H22P variant

Our ELDOR experiments on YF1 H22P with inverted signal response revealed a drastically altered LOV photosensor dimer interface. The N-terminal A’α helices that in the original YF1 are embraced by the two LOV photosensors (Fig. 1A) and that play important roles in signal propagation and modulation^19, 20^ are displaced and possibly unstructured in H22P. As unequivocally demonstrated by ELDOR distance measurements on photo-induced NSQ radicals of the FMN chromophores, the two LOV photosensors in H22P are much closer in distance than in the original YF1. The observed distance between the two NSQ perfectly agrees with a flush packing of the LOV domains against another via their β sheets. Strikingly, this altered quaternary structure largely corresponds to the arrangement in an earlier structure of the isolated *Bs*YtvA LOV domain that entirely lacked the A’α helices. Given its completely altered dimer interface, it is perplexing that the H22P variant still transduces light signals, and even more so, in inverted manner.

In the absence of a high-resolution structure of the H22P variant, we modelled light-induced structural transitions on the basis of the N-terminally truncated *Bs*YtvA LOV structure. Due to this approximation, it is challenging to extract reliable quantitative data, and the results should be considered qualitative in nature. Nonetheless, structural modelling constrained by the ELDOR data implied that the two LOV photosensors undergo a light-induced rotation and concomitant displacement of the Jα attachment sites that resemble the molecular response to light in YF1, even though the overall structure of the H22P variant is quite different from that of YF1. However, in marked difference to YF1, in the H22P variant light absorption led to an approach of the Jα anchor sites rather than a separation, consistent with the inverted signal response. Despite different initial structure and conformational transitions of YF1 and the H22P variant, similar forces are exerted on the Jα coiled coil and give rise to a common mode of signal propagation, albeit with inverted signal polarity. These findings exemplify the remarkable malleability and robustness of signal receptors which arguably promote rapid adaption to novel stimuli and rewiring of signalling pathways during evolution. Notably, the convergence of signal mechanisms appears to be a recurring theme in sensor histidine kinases^19, 36, 37^: For different sensor modules signal-induced responses as diverse as pivot, piston, rotation and association reactions have been identified, yet the regulation of histidine kinase activity could well follow a unifying mechanism^38^.

## Conclusion

By charting light-induced structural transitions within YF1, we find that signal transduction is predicated on tilting apart and rotation of the two LOV photosensor units that thereby exert stress on the attached Jα helices. Downstream signal transduction to the effector module is likely achieved by left-handed supercoiling of the Jα coiled coil and the directly connected DHp domain. Similar structural modes might apply to the large group of sensor histidine kinases, the signalling mechanism of which is still under vigorous debate^37^. As a case in point, structural and biochemical evidence for signal-induced rotary helical movements has been obtained for other sensor histidine kinases as well^34, 39, 40^.

The general motif of signal transduction evidenced in YF1 is conserved in the H22P variant despite drastically altered dimerization interface; intriguingly, the response to light of H22P is inverted at both the structural and functional levels. In conjunction with the high-resolution structure of dark-adapted YF1, our data provide valuable benchmarks for molecular dynamics (MD) simulations. Although individual LOV photosensors share a common protein fold and photochemistry, they respond to light absorption by distinct structural transitions, including unfolding of ancillary helices, association/dissociation and dimer rearrangements as presently^15^. Building on and extending previous MD studies^41^, it will be interesting to investigate how light-promoted protonation of the flavin N5 atom, be it by formation of the thioadduct, be it by reduction to the NSQ radical state^12^, is coupled to these divergent modes of signal propagation. In a similar vein, our present data inform the engineering of sensory photoreceptors on the basis of the *Bs*YtvA and related LOV domains. The now available knowledge of the light-induced structural transitions in this photosensor enables its functional coupling to desired effector modules. However, as the amplitude of these transitions is small, successful engineering will likely depend on particularly precise and finely calibrated joining of LOV photosensor and effector modules.

## Materials and Methods

Site-specific cysteine replacements were introduced into YF1 via the QuikChange protocol (Invitrogen, Life Technologies) within both the pET-41a expression construct and the pDusk-*myc*-*Ds*Red reporter plasmid^19^. Light-dependent signal transduction of YF1 variants was assessed in the pDusk reporter context^19^. YF1 variants were expressed ﻿in CmpX13 cells^42^ and purified as described^19^. Labelling was conducted with 10-fold molar excess of MTS. Light-dependent catalysis of MTS-labelled YF1 variants was assessed *in vitro* as described^12^. For EPR measurements, buffer was exchanged to deuterated HEPES supplemented with 50% (v/v) per-deuterated glycerol. Samples were kept in darkness or were illuminated for 5 min at 450 nm and were rapidly cooled in liquid nitrogen. Pulsed ELDOR spectra were recorded using the DEER sequence^22, 23^ Experiments were performed at 40 K in X- and 50 K in Q-band. ELDOR data were evaluated using DeerAnalysis^43^. Label rotamer simulations were performed using MMM^44^ and MtsslWizard^45^. ENM was performed in MMM, RBD in mtsslDock^26^. Molecular graphics were prepared with PyMOL^46^. For details, see SI.

## Electronic supplementary material

Supplementary Movie 1

Supplementary Information
