# Supplementary material for: Blue-light reception through quaternary transitions
Source: Sci Rep. 2017 May 3;7:1385. doi: 10.1038/s41598-017-01497-7 (PMC5431215; doi:10.1038/s41598-017-01497-7)
Supplement: Supplementary file 2 — Supplementary Information [file 41598_2017_1497_MOESM2_ESM.pdf]

Blue-light reception through quaternary transitions

Christopher Engelhard[1,*], Ralph P. Diensthuber[2,*], Andreas Möglich[2,3,¶], Robert Bittl[1,¶]

[1]Fachbereich Physik, Institut für Experimentalphysik, Freie Universität Berlin, 14195 Berlin, Germany
[2]Biophysikalische Chemie, Institut für Biologie, Humboldt-Universität zu Berlin, 10115 Berlin, Germany
[3]Lehrstuhl für Biochemie, Universität Bayreuth, 95440 Bayreuth, Germany

[*]C.E. and R.P.D. contributed equally
[¶]Correspondence to andreas.moeglich@uni-bayreuth.de or robert.bittl@fu-berlin.de

Supplementary information

Materials and methods

Site-directed mutagenesis and MTS spin labelling

YF1 comprises only a single cysteine residue, namely the conserved residue 62 which is engaged in light-driven formation of the thioether bond with the FMN chromophore and which is sequestered in the protein interior. YF1 thus affords an essentially cysteine-free background. Therefore, single site-directed mutagenesis and labelling sufficed for introducing spin labels at selected positions into the homodimeric YF1 receptor within each monomer. Site-specific cysteine replacements were introduced into YF1 via the QuikChange protocol (Invitrogen, Life Technologies) within both the pET-41a-YF1 expression construct and the pDusk-*myc*-*Ds*Red reporter plasmid [20]. All plasmids were verified by DNA sequencing (LGC Genomics, Berlin) and transformed into *E. coli* CmpX13 cells [45]. Protein expression from the pET-41a-YF1 plasmids and purification were carried out as described [20]. Purified proteins were dialyzed into storage buffer (10 mM Tris/HCl pH 8.0, 10 mM NaCl, 10 % [w/v] glycerol) and kept at -80°C. For spin labelling 400-600 µM of each YF1 variant was mixed with a 10-fold molar excess of MTSSL [(1-Oxyl-2,2,5,5-tetramethylpyrroline-3-methyl) methanethiosulfonate] and for 14 h at 4°C. Excess MTS spin label was removed by centrifugation for 10 min at 4,800 rpm and dialysis against 10 mM Tris/HCl pH 8.0, 10 mM NaCl, 2 % (w/v) glycerol.

Functional assays

Even though the MTS spin label is fairly small in molecular size, *a priori* it cannot be ruled out that its attachment to the YF1 variants, or for that matter, mutagenesis of the above residues to cysteine negatively impact on enzymatic activity and regulation by light. As described in the main text, the selected variants were assessed in a three stage process: 1. Functional assessment of the impact of cysteine replacement; 2. functional assessment of the impact of MTS attachment; and 3. validation after mutagenesis and label attachment against the dark-adapted structure of YF1.

1. Validation of cysteine replacements

For the first stage, the impact of the cysteine replacements on YF1 activity and light response was assessed in the pDusk-*myc*-*Ds*Red background [20]. Briefly, this plasmid encodes YF1, *Bj*FixJ and the red-fluorescent reporter *Ds*Red under control of the *Bj*FixK2 promotor; When transformed into *Escherichia coli*, these components drive expression of *Ds*Red in blue-light-repressed fashion. For the original YF1, reporter fluorescence is elevated by a factor of ~ 10-15 in darkness over blue light. CmpX13 cultures harbouring this plasmid were incubated for 16-18 h at 37°C and 225 rpm either in darkness or under constant blue light (470 nm, 100 µW cm^{-2}). Optical density at 600 nm (OD_{600}) and *Ds*Red fluorescence (F) of the cultures were measured in 96-well µClear plates (Greiner Bio One) using a Tecan M200pro multimode microplate reader. Settings for excitation and emission were 554 ± 9 nm and 591 ± 20 nm, respectively. Values of F/OD_{600} were normalized to dark-grown, reference cultures carrying the original pDusk-*myc*-*Ds*Red plasmid and represent the mean ± s.d. of three biological replicates. Corresponding assays for the YF1 and YF1 H22P variants are shown in Suppl. Figs. S1A and S1C.

2. Validation after MTS attachment

For the second stage, the MTS-labelled YF1 variants were tested *in vitro* as reported [12]. To this end, YF1 and *Bj*FixJ were incubated in the presence of ATP with a fluorescently

labelled, double-stranded DNA oligonucleotide harbouring a phospho-FixJ binding site. In the dark, the original YF1 phosphorylated *Bj*FixJ which subsequently binds to the olignucleotideand thereby decreases its electrophoretic mobility through a gel matrix within an electric field [12]. Briefly, 250 nM of a given MTS-labelled YF1 variant was mixed with 30 µM *Bj*FixJ, 1 mM ATP and 1.25 µM of a double-stranded DNA substrate that comprised the high-affinity *Bj*FixJ binding site of the *Bj*FixK2 promotor and a rhodamine fluorescent label. Samples were incubated at 30°C for 30' in reaction buffer (10 mM HEPES/HCl pH 8.0, 80 mM KCl, 2.5 mM $MgCl_2$, 0.1 mM EDTA, 100 µg ml^{-1} BSA, 10% [v/v] glycerol, 4% [v/v] ethylene glycol, 10 mM Tris(2-carboxyethyl)phosphine); incubation was either carried out in darkness or under constant blue light (455 nm, 50 mW cm^{-2}). Samples were then run on a 6% (w/v) acrylamide gel in TBE buffer (89 mM Tris, 89 mM borate, 2 mM ethylenediaminetetraacetic acid), and rhodamine fluorescence was visualized with a Fujifilm image reader FLA 3000 (Fujifilm Holdings K. K.) using excitation and emission of 532 and 580 nm, respectively. The corresponding assays are shown in Suppl. Figures S1B and S1D.

Additionally, the light-dependent activity of two YF1 variants was tested using the above described protocol before MTS labelling in a buffer containing 50% glycerol as in the EPR samples. Both the original YF1 and the H22P construct harbouring the additional D115C amino acid replacement for spin labelling suffer no activity impairment by the increased glycerol concentration as shown in Fig. S1E.

3. Validation against the YF1 crystal structure

For the third stage, the expected distance distributions for each position were calculated using the 4GCZ crystal structure [20]. Distance calculations were performed with the computer programs MMM [43] and MtsslWizard [44] (Suppl. Fig. S4). By utilising two programs with different algorithms for MTS insertion into the crystal structure, selection of MTS rotamers from a pre-calculated library and direct scanning of the MTS conformational space for van-der-Waals clashes for MMM and MtsslWizard, respectively, systematic calculation errors are minimized.

Electron paramagnetic resonance spectroscopy

Experimental setup

For EPR measurements, buffer was exchanged to deuterated HEPES supplemented with 50% (v/v) per-deuterated glycerol (Sigma Aldrich, St. Louis, MO, USA). Light-adapted amples were illuminated using a 450 nm LED (Luxeon Lumiled, Phillips Lumileds, San Jose, CA, USA) for 5 min and rapidly frozen in liquid nitrogen.

The samples were measured in quartz tubes (QSIL GmbH, Ilmenau, Germany) with 3.9 mm/3,0 mm and 3.0 mm/2.0 mm (inner/outer) diameter for X-band and Q-band experiments, respectively. X-band pulsed ELDOR spectra were recorded using the DEER sequence [23,24], employing a 12 ns pump pulse and 32 ns probe pulse in X-band, and 12 ns pulses throughout in Q-band, with a shot repetition time of 4 µs. Pulse separation for the initial Hahn echo was 346 ns in X-band and 400 ns in Q-band. Pulses were set up with the pump pulse exciting the spectrum at the position of maximal intensity, with the probe pulses offset to the secondary maximum 70 MHz higher (X-band) and 100 MHz lower (Q-band). The X-Band setup consisted of a EPR spectrometer Bruker E680 with ELDOR microwave source E580-400U in conjunction with a dielectric-ring resonator Bruker EN4118X-MD5 (all from Bruker Biospin GmbH, Rheinstetten, Germany) and TWT microwave pulse amplifier Applied Systems Engineering Model 117X (Applied Systems Engineering Inc., Forth Worth, TX, USA). The Q-Band setup comprised a Bruker SpinJet AWG spectrometer, using an Applied Systems Engineering Model 187Ka TWT amplifier and a large-bandwidth cavity developed and built by E. Bordignon at ETH Zurich, Switzerland. Cryogenic temperatures were reached

using Oxford CF-935 cryostats (Oxford Instruments, Oxfordshire, UK) and Oxford ITC503 and MercuryITC temperature controllers for X- and Q-band, respectively.

Data analysis

All data analysis was performed in MatLab (The MathWorks Inc., Natick, MA, USA) and PyMol [46]. Background-corrected ELDOR data of spin-labelled variants was evaluated using Tikhonov regularisation as implemented DeerAnalysis [42] (Supp. Fig. S3 (YF1) and S7A-I (YF1 H22P). Background-corrected data of flavin-radical variants C62A and H22P:C62A were treated with a Hamming window and Fourier-transformed (Supp. Fig. S7J). The interspin distance was derived from the maxima of the dipolar spectrum.

EPR-constrained structural modelling

An elastic network model (ENM) as implemented in MMM [27] and a constrained rigid-body docking algorithm (RBD) as implemented in mtsslDock [28] were used to generate model structures with the centre distances of the dominant features in the ELDOR-derived $p(r)$ distributions weighted by the width of these distributions as experimental constraints. The distance constraints used for model building are summarized in table S1 for the original YF1 construct as well as YF1 H22P.

Structural modelling for YF1

For ENM, the YF1 structure was truncated at residue I126 at the base of the Jα linker, since due to lack of constraints in this linker or the DHp and CA domains, transversal modes of the entire dimer along the linker dominated the fit. For RBD, the YF1 structure was truncated at H145 and thus included the Jα linker; two LOV monomers were docked onto another using as constraints the ELDOR data and additional constraints for residues within the Jα linker derived from the 4GCZ crystal structure. In case of the P87C position, it is not certain whether the discrepancy between measured and simulated distance distributions was due to real differences in the YF1 structure between crystal and solution, or whether it reflected a structural perturbation induced by spin labelling. We hence performed separate modelling runs with the constraints for P87C either included or excluded. The resulting 8 model structures are shown in Suppl. Figs. S5A-D.

Structural modelling for YF1 H22P

YF1 H22P simulations were based on the *Bs*YtvA structure 2PR5 [22]. For ENM, the structure was again cut at residue I126, analogous to the YF1 models. RBD was attempted but failed to produce realistic docking models, most likely due to differences in the structure of *Bs*YtvA lacking the N-terminal helices and YF1 H22P. As the measured P87C distance again a large deviation from the distance derived from the *Bs*YtvA structure, separate modelling runs with this constraint either included or excluded were performed. The resulting model structures for the dark and the light adapted states are shown in Suppl. Figs. S8A and S8B.

Tables

Supplementary Table S1: EPR-derived distance constrains for YF1 and YF1 H22P in the dark and light state, respectively.

		dark		light	
	**residue pair**	**distance / nm**	**width / nm**	**distance / nm**	**width / nm**
**YF1**	**Q44-Q44**	2.84	0.20	2.84	0.20
**YF1**	**E55-E55**	5.03	0.20	5.03	0.20
**YF1**	**D71-D71**	4.73	0.40	4.98	0.40
**YF1**	**D76-D76**	6.21	0.30	6.33	0.30
**YF1**	**N84-N84**	6.08	0.30	6.22	0.30
**YF1**	**P87-P87**	2.72	0.40	3.05	0.40
**YF1**	**M101-M101**	3.61	0.30	3.73	0.30
**YF1**	**D115-D115**	4.00	1.00	4.00	1.00
**YF1 H22P**	**E55-E55**	4.50	1.00	4.35	1.00
**YF1 H22P**	**D71-D71**	5.58	0.30	5.65	0.30
**YF1 H22P**	**D76-D76**	5.68	0.30	5.77	0.30
**YF1 H22P**	**N84-N84**	5.07	0.30	5.13	0.30
**YF1 H22P**	**P87-P87**	1.84	0.20	1.84	0.20
**YF1 H22P**	**Q93-Q93**	3.73	0.40	4.35	0.60
**YF1 H22P**	**M101-M101**	4.23	0.40	3.35	0.40
**YF1 H22P**	**D115-D115**	1.98	0.50	2.25	0.50

Figures

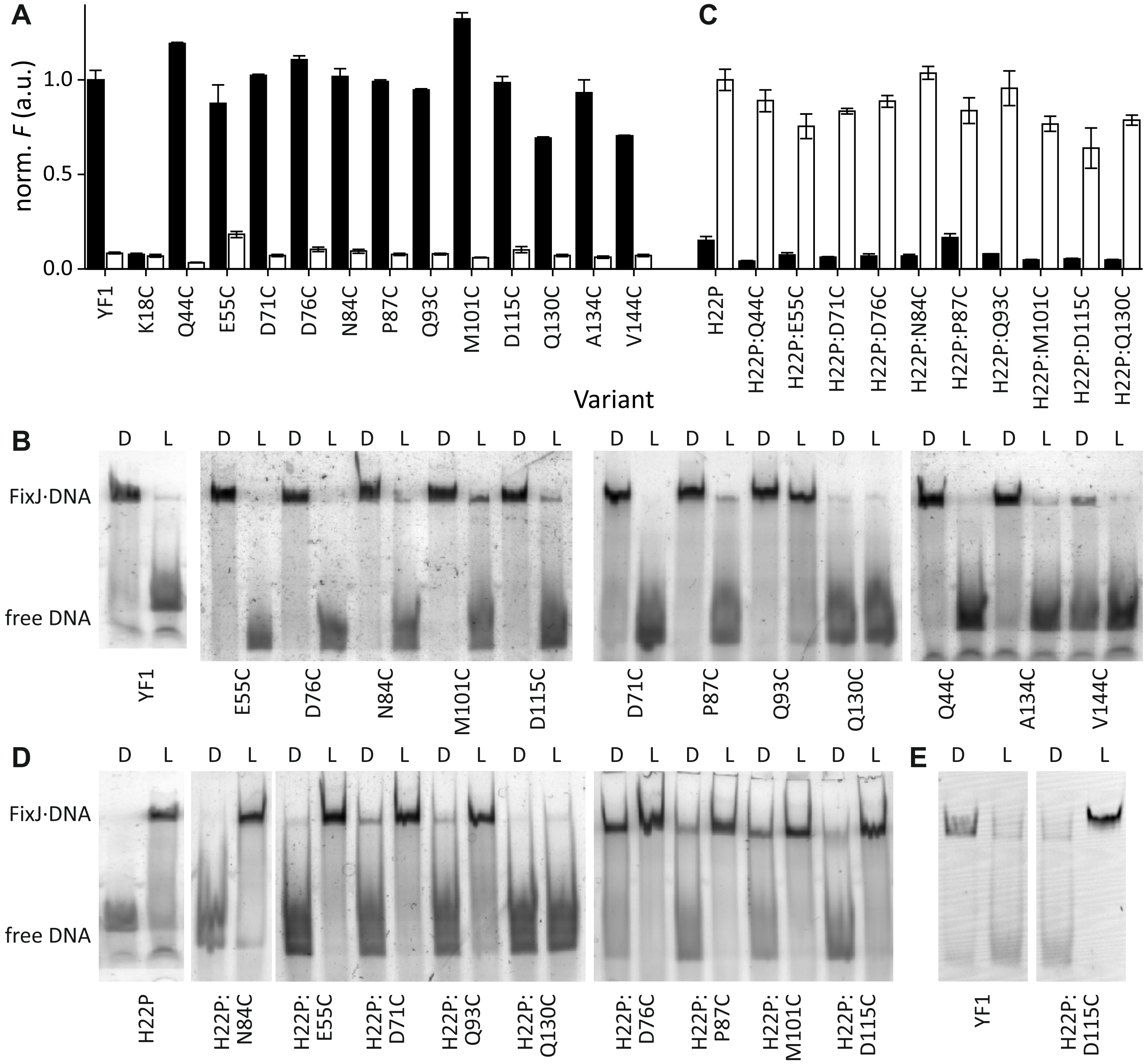

Supplementary Figure S1: Functional assays of YF1 variants without and with attached spin label MTS. (**A**) With the exception of YF1 K18C, which lacks any light response, all variants excluding the H22P mutation show YF1-like light-repressed activity. (**B**) After labelling with MTS, this functionality is fully or mostly retained in all tested variants except YF1 Q93C, Q130C, V144C. (**C**) All variants including the H22P mutation show an inverted, light-activated response. (**D**) After labelling with MTS, this functionality is fully or mostly retained in all tested variants except YF1 H22P:Q130C. (**E**) The presence of 50 % glycerol, as used in the EPR measurements, does not impair light-dependent activity, neither in the YF1 nor in the YF1 H22P context.

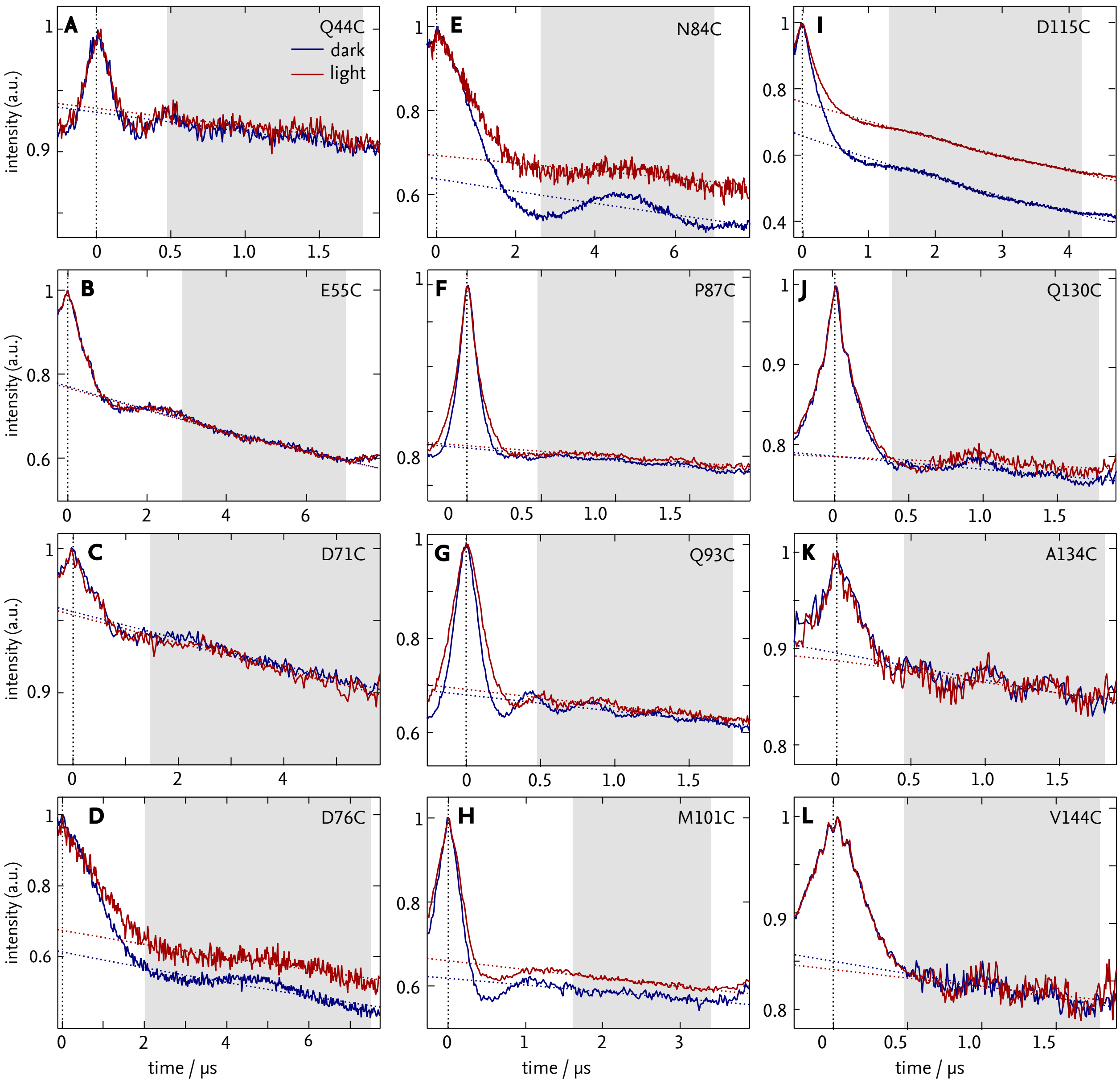

Supplementary Figure S2: Raw ELDOR data of spin labelled YF1 variants. Data for Q130C and V144C are the result of combining a short high resolution and low noise time trace with a less resolved longer timetrace to improve background correction. The areas used for background correction are shown in gray, the resulting fitted backgrounds are shown as dotted lines.

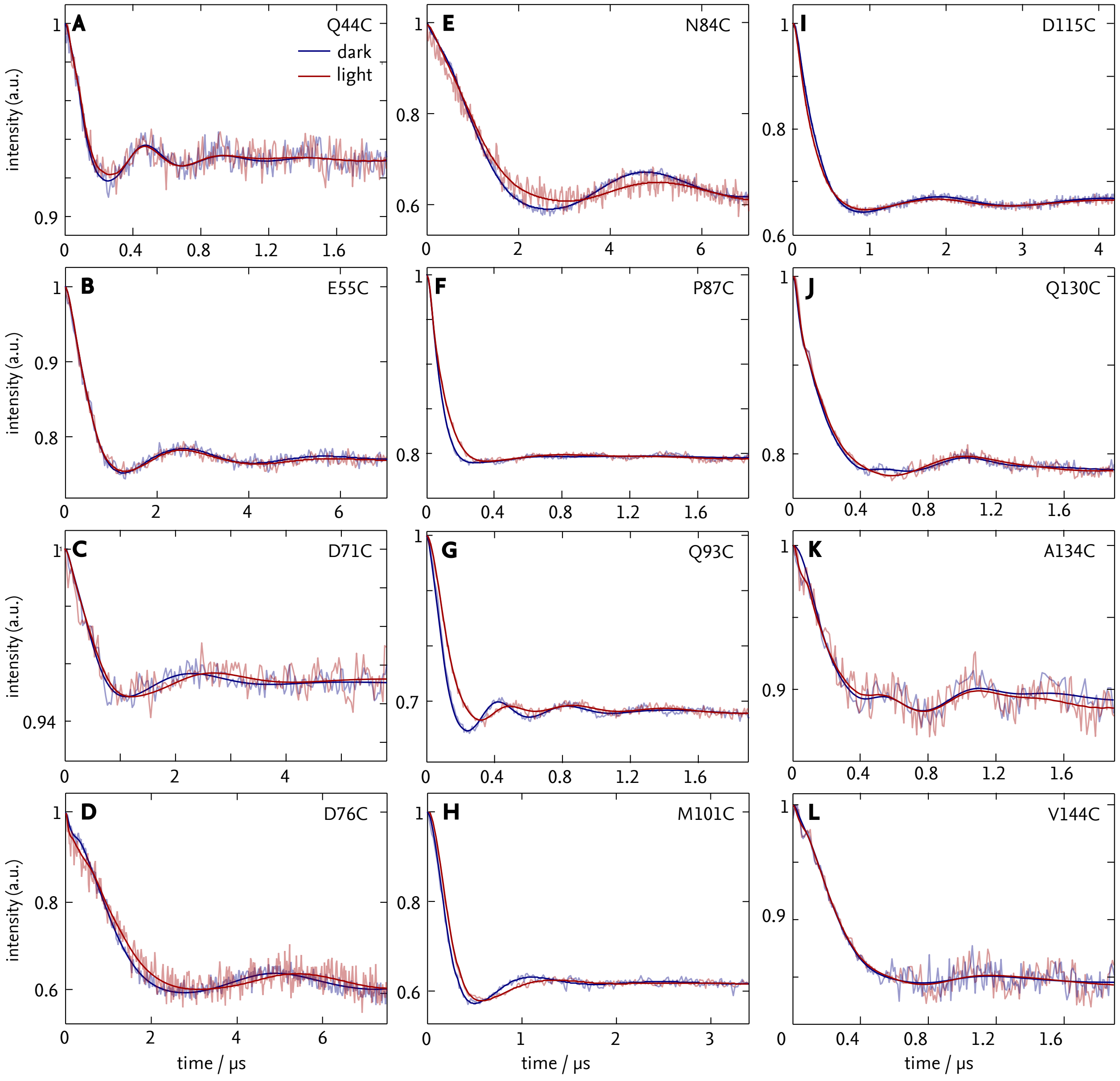

Supplementary Figure S3: Fit of ELDOR data of spin labelled YF1 variants. Background-corrected ELDOR data of spin labelled YF1 variants (shaded) with fit derived from Tikhonov regularisation (solid). The modulation depth of the light-adapted state data has, where necessary, been scaled to match that of the dark state data.

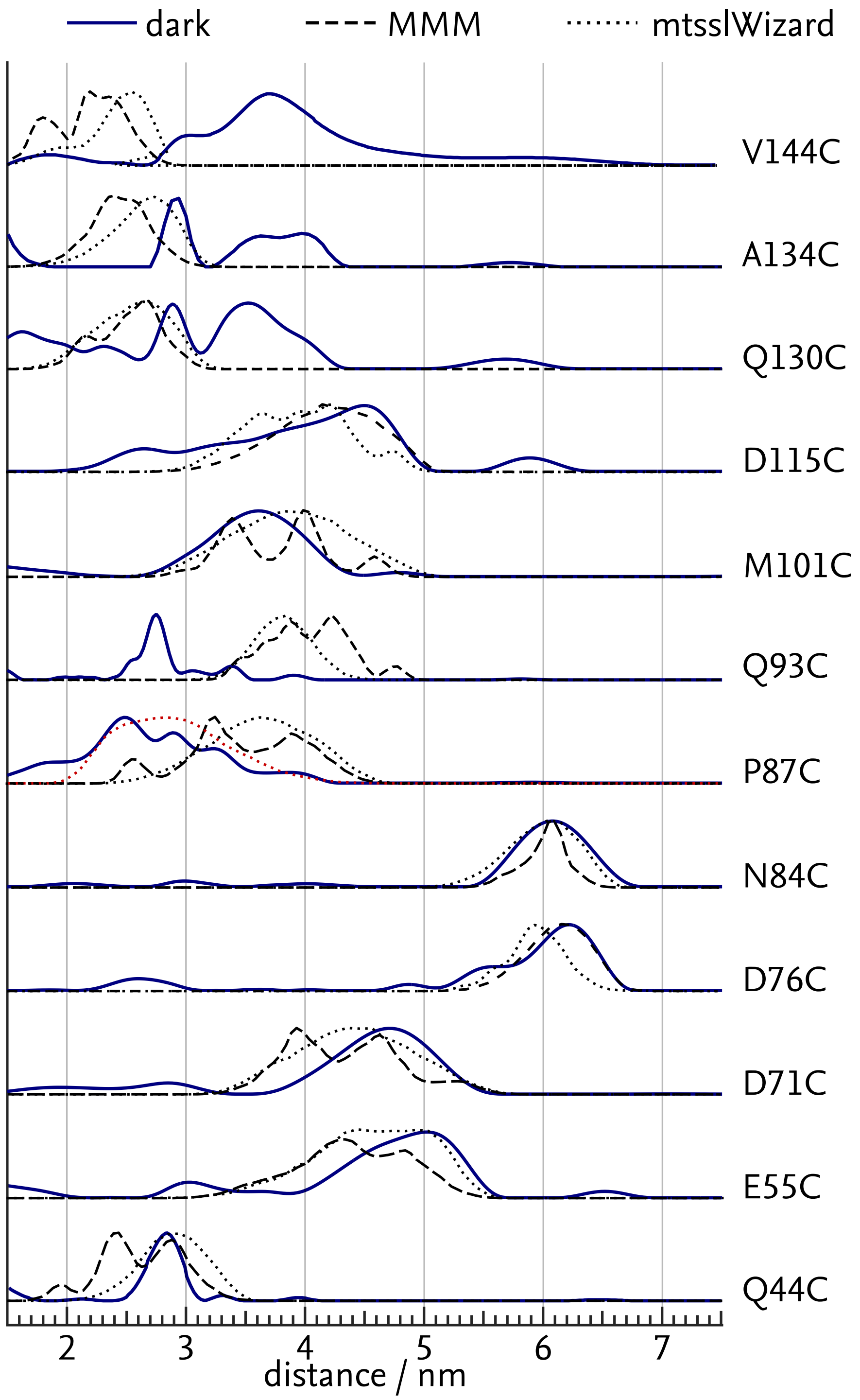

Supplementary Figure S4: Distances observed for YF1 in the dark state (blue) compared to calculated distances (black). Calculations are based on the YF1 crystal structure 4GCZ [20] using MMM [43] (dashed) and MtsslWizard [44] (dotted). The three linker positions V144C, A134C and Q130C show significantly larger distances than calculated, the measured distance for Q93C is significantly too short. For P87C, only a subset of the simulated spin label rotamers (red dotted line) represents the measured distribution. For all other variants the measured distances match the calculated ones.

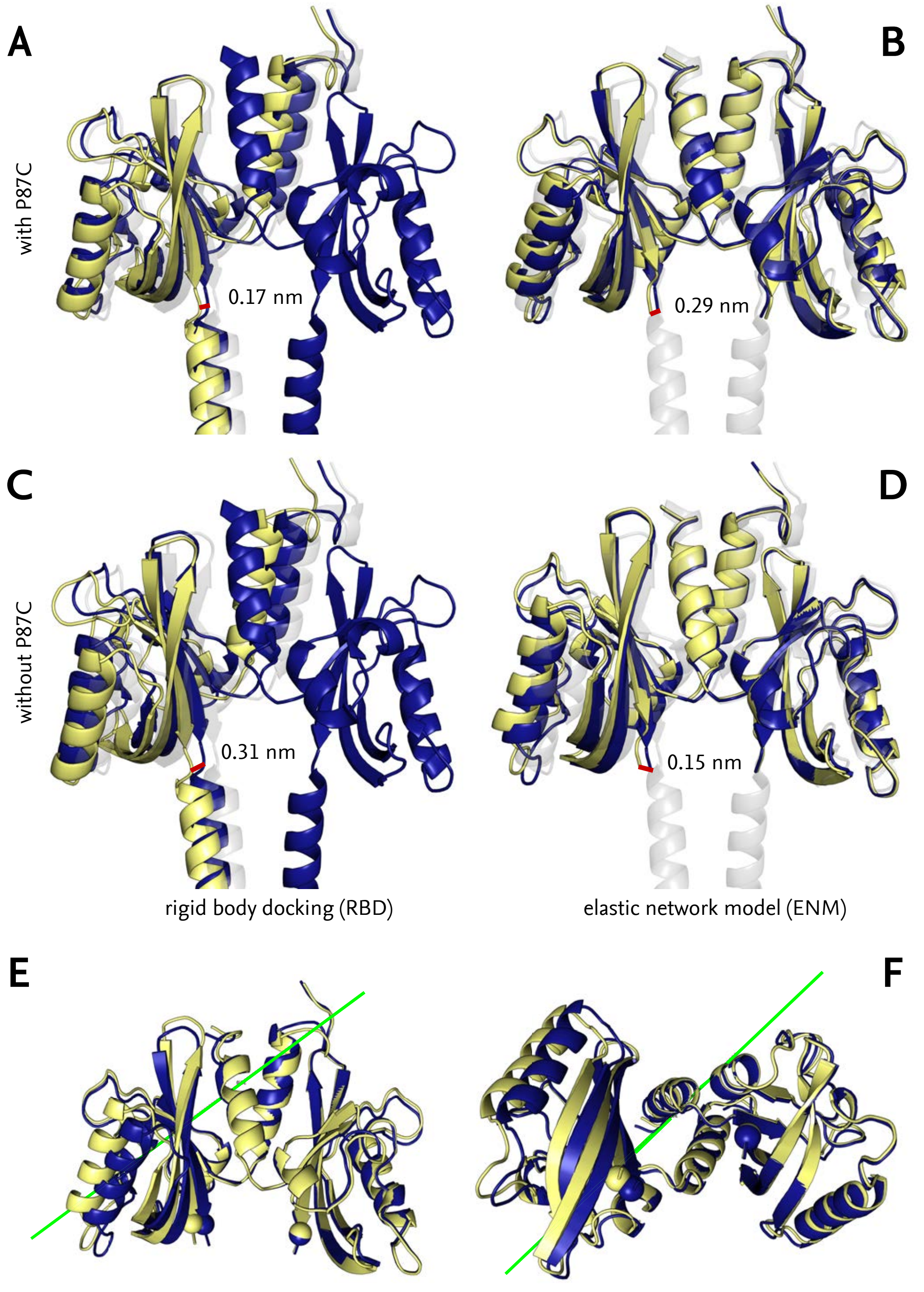

Supplementary Figure S5: Model structures for YF1 in the dark (blue) and light (yellow) state. Model structures were generated using (**A**) RBD with the P87C distance included, (**B**) ENM with P87C, (**C**) RBD without P87C, (**D**) ENM without P87C. All models show a similar movement motif in which the connection points of the linker are shifted by between 0.15 nm and 0.31 nm (measured between the Cα atoms of residues I126). (**E-F**) Alignment after transition from dark to light state structure described as a screw-rotation movement, i.e. rotation around and translation along a single axis, viewed from the side (**E**) and bottom (**F**) of the LOV domains. The rotation/translation axis is shown in green.

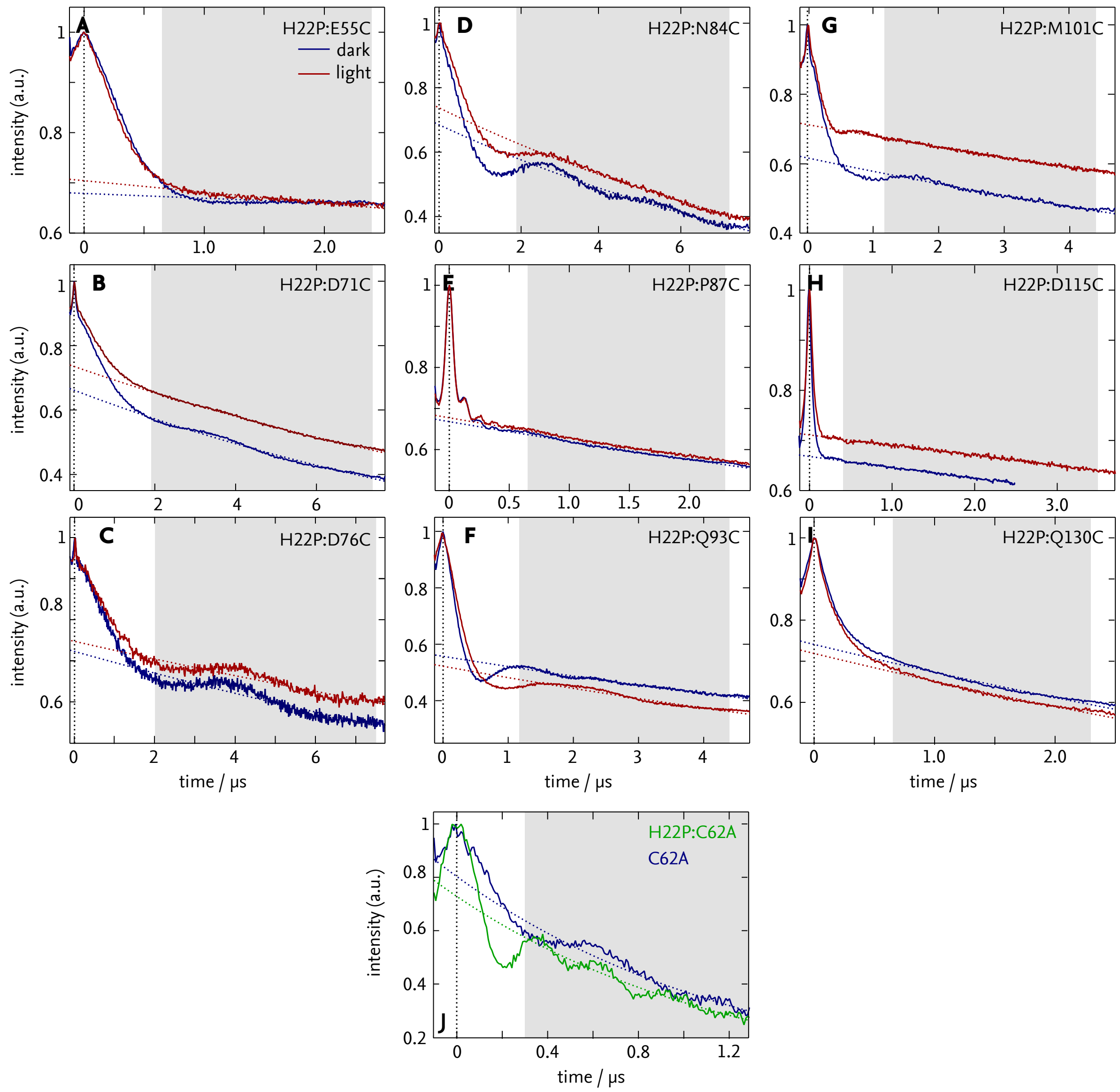

Supplementary Figure S6: Raw ELDOR data of spin labelled YF1 H22P and of YF1 C62A variants. The areas used for background correction are shown in gray, the resulting fitted backgrounds are shown as dotted lines.

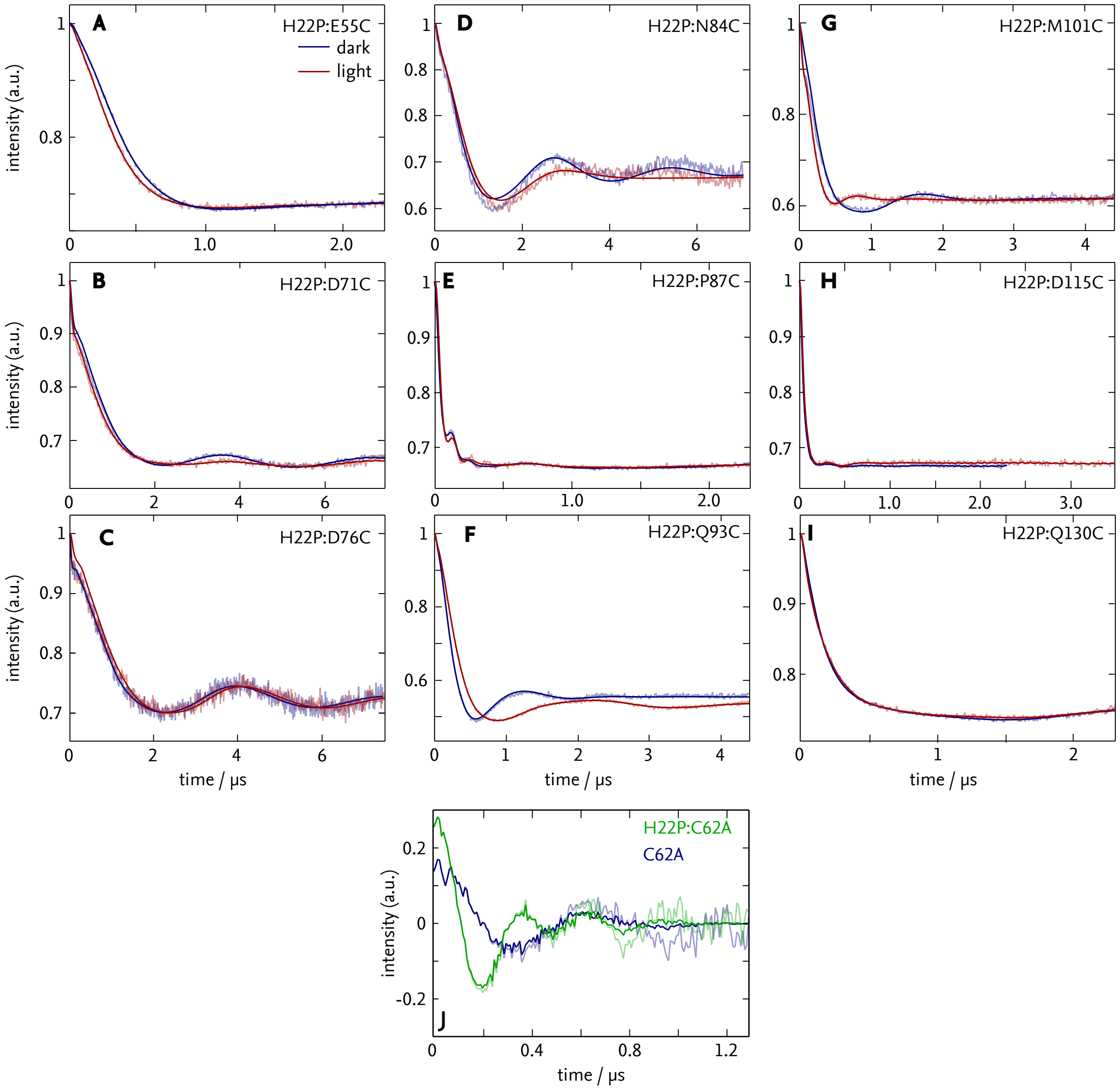

Supplementary Figure S7: Fits of ELDOR data of spin labelled YF1 H22P and of YF1 C62A variants. (**A-I**) Background-corrected ELDOR data of spin labelled YF1 H22P variants (shaded) with fit derived from Tikhonov regularisation (solid). The modulation depth of the light-adapted state data has, where necessary, been scaled to match that of the dark state data. (**J**) Background-corrected ELDOR data of YF1 C62A and H22P C62A (shaded colors) and after additional application of a Hamming window (solid colors) for Fourier transform.

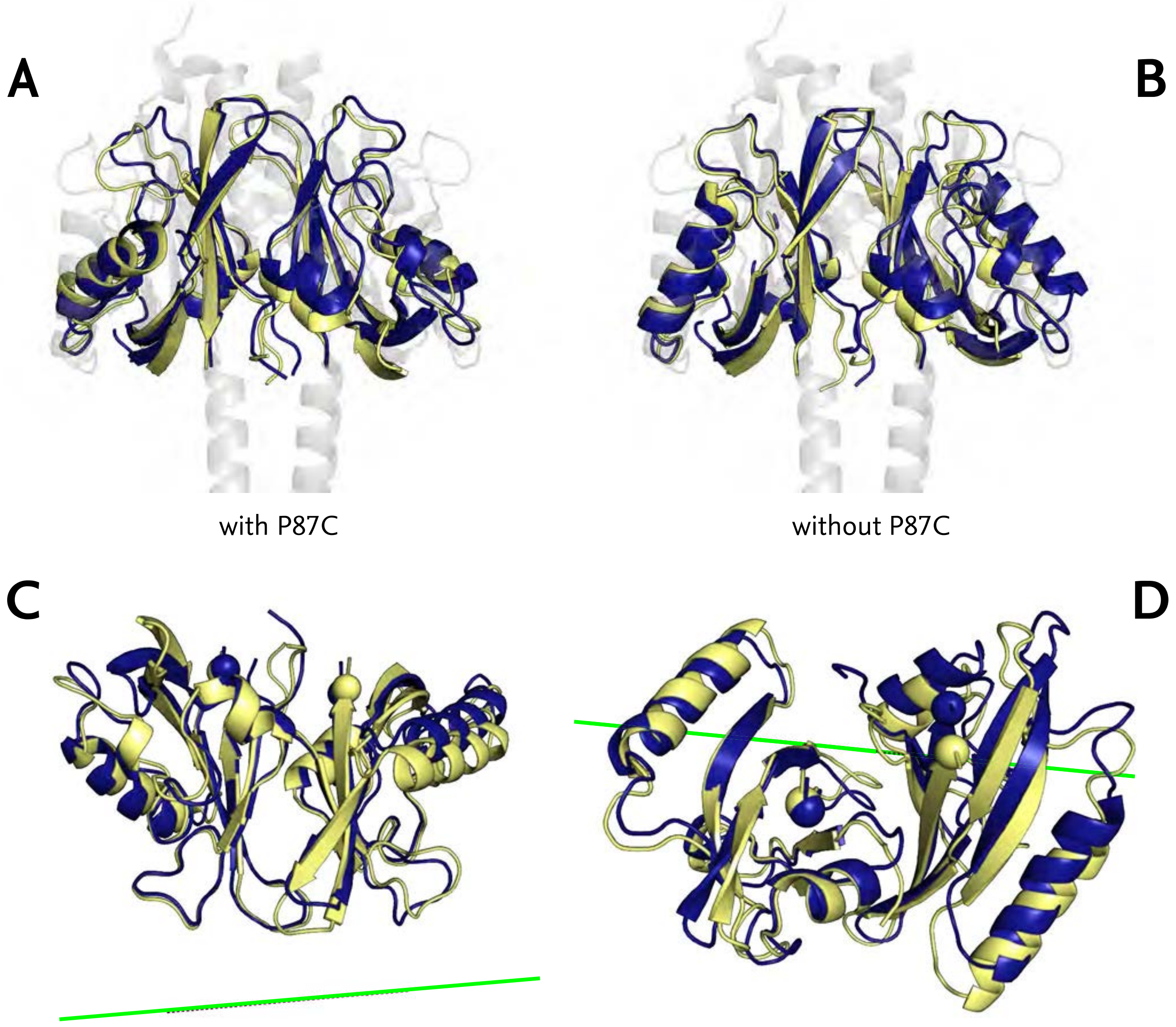

Supplementary Figure S8: Model structures for YF1 H22P in the dark (blue) and light (yellow) state based on the *Bs*YtvA structure. Structures were generated using ENM with (**A**) and without (**B**) the P98C constraint included. While the domain interface is very different from YF1 due to the lack of N-terminal helices, again the data predicts a shift of the connection points to the Linker region. While the two models differ significantly in the vicinity of P87C to accommodate the measured distances, the predicted movement of the connections to the linker are very similar. (**C-D**) Alignment after transition from dark to light state structure described as a screw-rotation movement, viewed from the side (**C**) and bottom (**D**) of the LOV domains. The rotation/translation axis is shown in green.

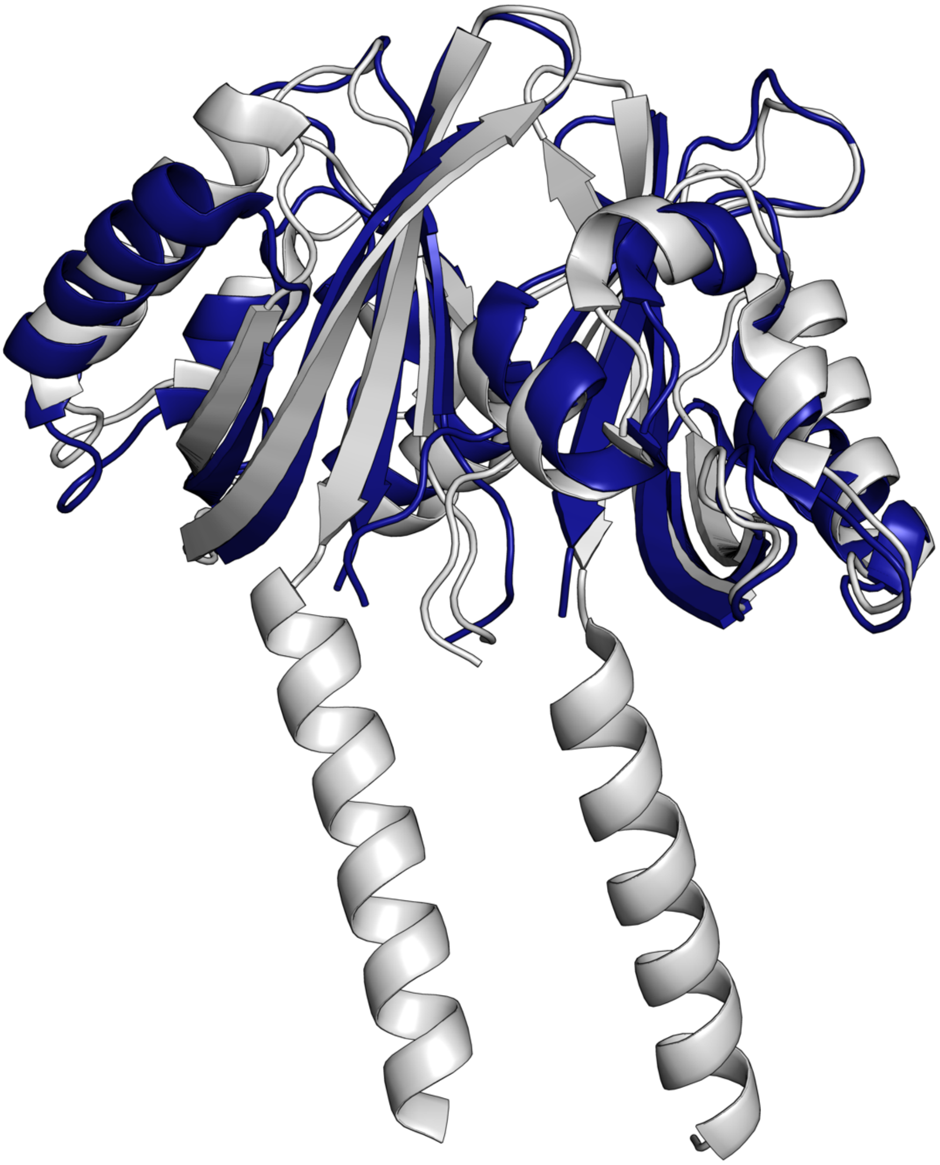

Supplementary Figure S9: Model structure for YF1 H22P in the dark (blue) and the *Bs*YtvA structure 2PR5 (grey). The model structure for YF1 H22P in the dark generated using ENM without the P98C constraint and used in figs. 3C, S8B-D is shown in comparison to the *Bs*YtvA crystal structure 2PR5.

Still image of Supplementary Movie MS1

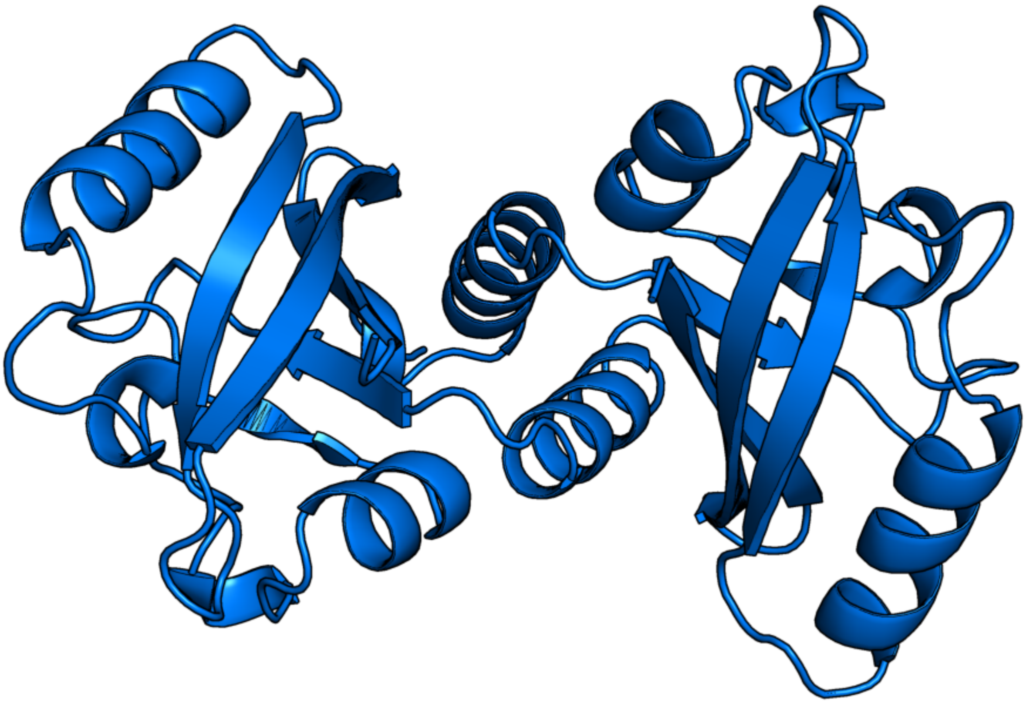

Supplementary Movie 1: Animation of the transition from the dark-adapted to the light-adapted state. The motif of an outward tilting of the two LOV domains can be clearly seen in the dark-light transition derived from EPR distance constraints.
